# Previous Intensive Care Unit Experience Improves Family Satisfaction With Decision‐Making: A Survey

**DOI:** 10.1111/nicc.70403

**Published:** 2026-02-25

**Authors:** Pei‐Yi Wang, Mu‐Hsing Ho, Chia‐Chin Lin

**Affiliations:** ^1^ Department of Nursing National Taiwan University Hospital, National Taiwan University College of Medicine Taipei Taiwan; ^2^ School of Nursing, Li Ka Shing Faculty of Medicine The University of Hong Kong Pokfulam Hong Kong; ^3^ Alice Ho Miu Ling Nethersole Charity Foundation Professor in Nursing Tai Po Hong Kong

**Keywords:** decision‐making, family members, intensive care unit, personal satisfaction, satisfaction

## Abstract

**Background:**

Family members often encounter decision‐making challenges when providing care. Family satisfaction is an indicator used to measure the quality of intensive care.

**Aim:**

This study measured satisfaction with decision‐making among family members of patients with critical illness in the intensive care unit (ICU).

**Study Design:**

A cross‐sectional descriptive study was conducted with family members of ICU patients at a medical centre in southern Taiwan. We developed a traditional Chinese version of the 24‐Item Family Satisfaction in the ICU (FS‐ICU 24) questionnaire and surveyed the family members of ICU patients in Taiwan. The factors influencing family satisfaction with ICU care were analysed using the independent *t*‐test and Pearson's correlation.

**Results:**

In total, 100 family members participated in this study. The traditional Chinese version of the FS‐ICU 24 exhibited excellent reliability and validity. Each item score was converted to a 0–100 scale, with higher scores reflecting greater family satisfaction. The participants' overall satisfaction score with ICU care (85.7 ± 15.4) and their satisfaction with decision‐making (84.5 ± 13.8). Moreover, decision‐making satisfaction was significantly higher among participants who previously experienced having family members admitted to the ICU than among those who had not (*p* = 0.017).

**Conclusions:**

The findings suggest that intensivists and nurses should proactively share information with patients' family members, particularly those without previous experience in the ICU. This approach can help enhance the quality of supportive care and improve family‐centred care in the ICU.

**Relevance to Clinical Practice:**

ICU healthcare teams should prioritise clear communication and active interaction with patients and their families, helping families feel more reassured about the treatments their loved ones receive.

## Impact Statements

- What is known about the topic
  ○ Family satisfaction in the ICU is an indicator of the quality of decision‐making and care.
  ○ Decision‐making is a complex process that aims to meet the needs of both patients and families.
  ○ Previous ICU experience may increase family members’ desire to participate in patient care and reduce perceived stress.
- What this paper adds
  ○ The validity and reliability of the traditional Chinese version of the FS‐ICU 24 were examined.
  ○ Family satisfaction with care was positively correlated with satisfaction in decision‐making for critically ill patients.
  ○ The healthcare team must communicate carefully and attentively with family members who lack ICU experience.

## 1 Introduction and Background

The experience, opinions and satisfaction of patients and their families are essential indicators of medical care quality [1]. Family members of intensive care unit (ICU) patients often encounter decision‐making challenges in terms of medical treatments and healthcare services, which may result in the lack of representation of their preferences and opinions [2, 3, 4]. It may be valuable to recognise that involving families in decision‐making is just one of several important strategies for supporting these discussions. This can be a great burden for the family, particularly in end‐of‐life decision‐making [4]. The involvement of families in end‐of‐life decision‐making should be properly organised within the ICU [4, 5].

Satisfaction is another indicator for measuring the quality of medical care and shared decision‐making, which refers to the subjective cognition and evaluation of patients or family members regarding services provided by a medical institution [6, 7]. Most patients admitted to the ICU often feel pressured and anxious because of the unfamiliar environment, disease severity, and loss of their life routine and ability to make decisions regarding medical treatments and healthcare services [8]. Furthermore, the family members' anxiety is aggravated by their inability to understand the patient's condition and make decisions and by their concern for the patient [9, 10]. Nevertheless, previous ICU experience may increase the desire of family members to participate in patient care and decrease perceived stress [11, 12].

However, the satisfaction levels of family members with prior ICU experience have not yet been investigated. In this study, the *24‐Item Family Satisfaction in the ICU* (FS‐ICU 24) questionnaire was translated into traditional Chinese, and the satisfaction of family members of ICU patients with the decision‐making process and care provision in the ICU was evaluated.

## 2 Design and Methods

### 2.1 Study Design and Participants

In this cross‐sectional descriptive study, a questionnaire survey was administered to the family members of ICU patients at a medical centre in Taiwan, where ICU family visiting hours are scheduled twice daily, with each visit limited to 30 min. During these visits, the attending physician or nursing staff typically explain the patient's current condition and treatment plan to family members. The participants were recruited between 1 January 2018 and 31 December 2018. The inclusion criteria were as follows: (1) patient's main decision‐maker; (2) family member of an ICU patient who has been on a mechanical ventilator for > 48 h; (3) aged > 18 years; (4) an educational level of high school or higher; and (5) can speak Mandarin or Taiwanese. The exclusion criteria were as follows: (1) potential medical disputes and (2) mental illness or cognitive impairment, as reported by the ICU staff. The sample size was determined using G*Power version 3.1, based on pilot study results. A minimum of 84 participants was required (α = 0.05, power = 0.95, effect size = 0.8). Considering a 20% attrition rate, the final estimated sample size was 105 participants.

### 2.2 Data Collection

The demographics of the family members (i.e., age, sex, relationship with the patient, previous experience of having a family member in the ICU, and living with the patient or not) and ICU patients (i.e., sex, ICU type, age, Acute Physiology and Chronic Health Evaluation [APACHE] II score, mechanical ventilation, DNR order, comorbidity, ward before ICU, type of admission, ventilator days, ICU length of stay and ICU survival [died in the ICU or not]) were collected.

### 2.3 Traditional Chinese Version of the FS‐ICU 24

The FS‐ICU was developed by the End‐of‐Life Network (CARENET) research team in Canada in 2001 [13]. Wall et al. performed factor analysis to modify the questionnaire into a short form, namely, FS‐ICU 24 [14]. In this modified version, the items of the longer version that corresponded to the family satisfaction with care and family satisfaction with decision‐making sections were reorganised according to their factor loadings. Ultimately, the questionnaire was finalised to contain 24 items. The first section of the questionnaire (satisfaction with care) comprised 14 items, and the second section (satisfaction with decision‐making around care of critically ill patients) comprised 10 items. The rating method of the questionnaire was similar to that of the long‐version FS‐ICU—that is, the score of each item was 0–100 after conversion, with a higher score indicating greater family satisfaction.

To develop the traditional Chinese version of the FS‐ICU 24, the psychometric properties were examined. After obtaining the consent of the original author, two English language experts were invited to perform forward–backward translations. The FS‐ICU 24 was translated from English to Chinese using the forward–backward translation method, in accordance with Brislin's guidelines, which outline detailed procedures for both translation and back‐translation [15]. The reliability and validity of the traditional Chinese version of the questionnaire were then tested. The translated version was first tested for its content validity index (CVI), which was determined by four experts and scholars with experience in ICU care and administration. The criteria for content validity were as follows: (1) representativeness of the content; (2) clarity of the item; (3) item structure; (4) comprehensiveness of the measure; and (5) consistency between the translated and backward‐translated versions [16].

### 2.4 Statistical Analysis

The categorical variables of the descriptive statistics are expressed as frequencies and percentages, whereas the continuous variables are expressed as means and standard deviations. Factors associated with family satisfaction with ICU were analysed using the independent *t*‐test and Pearson's correlation. The aforementioned statistical analyses were performed using SAS (version 9.4; SAS Inc., Cary, NC, USA). *p* values < 0.05 were used to indicate statistical significance.

### 2.5 Ethical Considerations

The study protocol was approved by the Institutional Review Board (TMU‐JIRB no.: 201208028, Approval Date: 01/09/2012 and NCKUH‐IRB no.: A‐ER‐106‐291, Approval Date: 01/01/2018). Written informed consent was obtained from the participants after receiving an explanation of the purpose of the study and agreeing to participate.

## 3 Results

### 3.1 Validity and Reliability of the Traditional Chinese Version of the FS‐ICU 24 Questionnaire

The overall CVI of the traditional Chinese version of the FS‐ICU 24 was 0.93. This questionnaire was then used to conduct a pilot study in an ICU in northern Taiwan. Twenty‐four family members participated in the pilot test. Regarding internal consistency and subscale‐total correlation, the Cronbach α values were 0.95 and 0.83 for Satisfaction with Care and Satisfaction with Decision‐Making Around Care of Critically Ill Patients, respectively. Furthermore, the correlation coefficients were 0.96 and 0.91, respectively, indicating excellent reliability (*p* < 0.0001). In this study, the validity and reliability of the traditional Chinese version of the FS‐ICU 24 were established.

### 3.2 Demographic Characteristics of ICU Patients and Their Family Members

In total, 105 participants were recruited from the ICU of a medical centre in southern Taiwan between 1 January 2018 and 31 December 2018, and 100 valid questionnaire responses were collected, demonstrating a valid response rate of 95.2%. Tables 1 and 2 present the demographics of the ICU patients and their family members, respectively. Most participants were women (59%), with an average age of 46.0 ± 12.4 years. Of the participants, 61.6% were children of the patients, 13.1% were the patients' spouses, 57.6% lived with the patients, and 54.1% had no previous experience of family members admitted to the ICU. The participants' total scores for Satisfaction with Care and Satisfaction with Decision‐Making Around Care of Critically Ill Patients were 85.4 ± 15.4 and 84.5 ± 18.3, respectively (Table 1).

**TABLE 1:** Demographic characteristics of the family members in the ICU (*N* = 100).

Variables	*n* (%)
Sex	
Male	41 (41)
Female	59 (59)
Age (mean ± SD)	46.0 ± 12.4
Relationship with patient	
Children	61 (61.6)
Spouse	13 (13.1)
Parents	10 (10.1)
Siblings	5 (5.1)
Partner	3 (3.0)
Others	7 (7.1)
Previous ICU experience	
No	53 (54.1)
Yes	45 (45.9)
Living with the patient	
Yes	57 (57.6)
No	42 (42.4)

Abbreviations: ICU, intensive care unit; SD, standard deviation.

**TABLE 2:** Demographic characteristics of the patients in the ICU (*N* = 100).

Variables	*n* (%)
Sex	
Male	67 (67.0)
Female	33 (33.0)
Types of ICU	
MICU	55 (55.0)
SICU	45 (45.0)
Age (mean ± SD)	67.4 ± 13.9
APACHE II (mean ± SD)	21.7 ± 7.70
Mechanical ventilation	
Invasive	98 (98.0)
Noninvasive	2 (2.0)
DNR consent form	
No	65 (65.0)
Yes	35 (35.0)
Comorbidity	
Yes	75 (75.0)
No	25 (25.0)
Ward before ICU	
Emergency room	66 (66.0)
General ward	34 (34.0)
Type of admission	
Elective	63 (63.0)
Operation	21 (21.0)
CPR	16 (16.0)
Ventilator days	24.1 ± 22.7
ICU LOS	27.9 ± 24.8
ICU survival	
Died	14 (14.0)

Abbreviations: APACHE, Acute Physiology and Chronic Health Evaluation; CPR, cardiopulmonary resuscitation; DNR, do‐not‐resuscitate; ICU, intensive care unit; LOS, length of stay; MICU, medical intensive care unit; SD, standard deviation; SICU, surgical intensive care unit.

Most patients were men (67%) and were admitted to the medical ICU (55%); their average age was 67.4 ± 13.9 years. The average APACHE II score upon admission to the ICU was 21.7 ± 7.70. Of the patients, 75% had comorbidities, and 66% were transferred from the emergency department to the ICU. Furthermore, 98% were placed on invasive mechanical ventilation at an average duration of 24.1 ± 22.7 days. The average length of ICU stay was 27.9 ± 24.8 days. Of the patients, 65% did not provide a signed DNR consent form. Before ICU admission, 21% underwent urgent surgery, and 16% of patients underwent cardiopulmonary resuscitation. Approximately 86% of patients were alive when they left the ICU (Table 2).

### 3.3 Family Satisfaction With Care and Decision‐Making in the ICU

**FIGURE 1:**
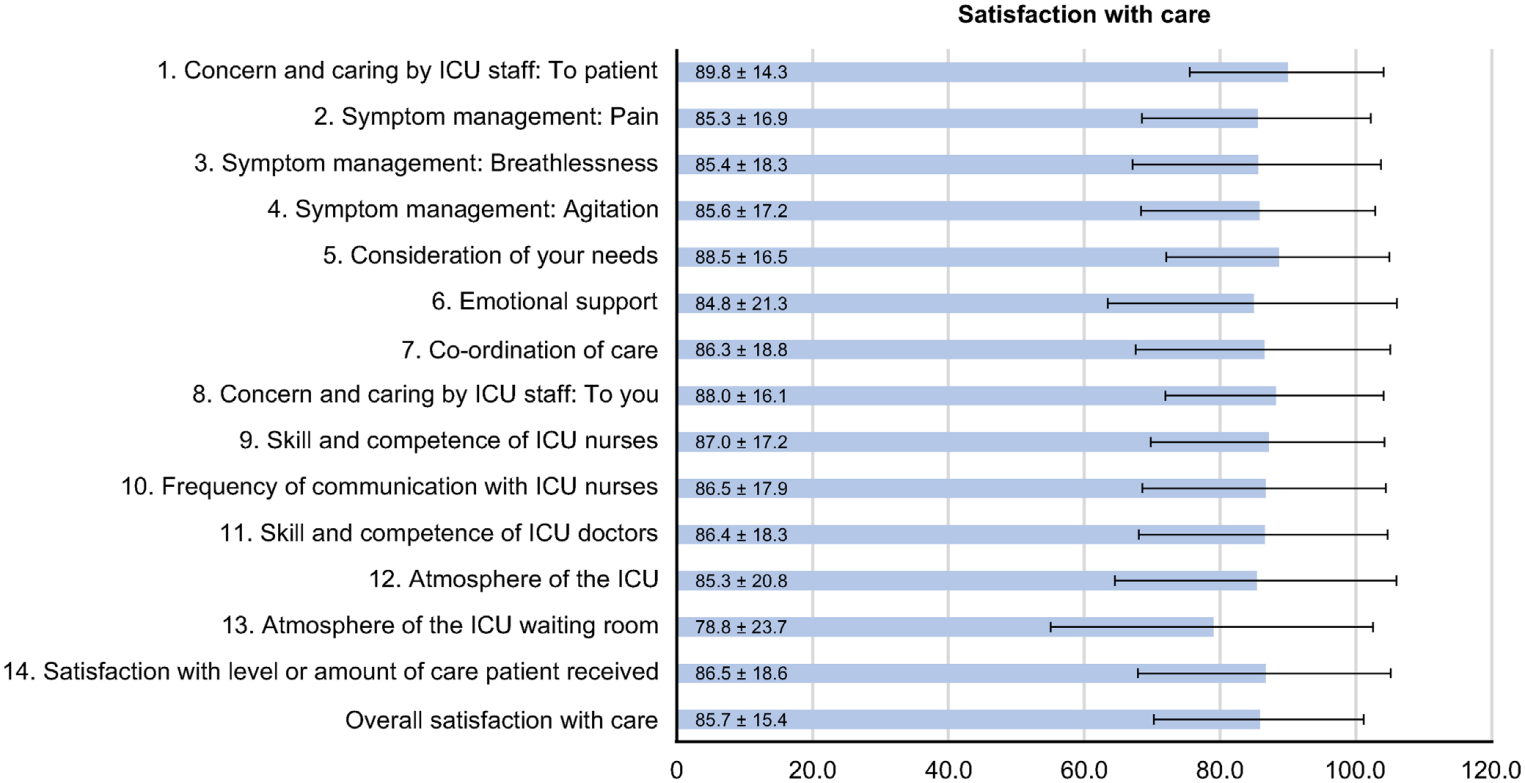
Mean scores and standard deviations for items on the *Family Satisfaction with Care* subscale of the FS‐ICU 24.

**FIGURE 2:**
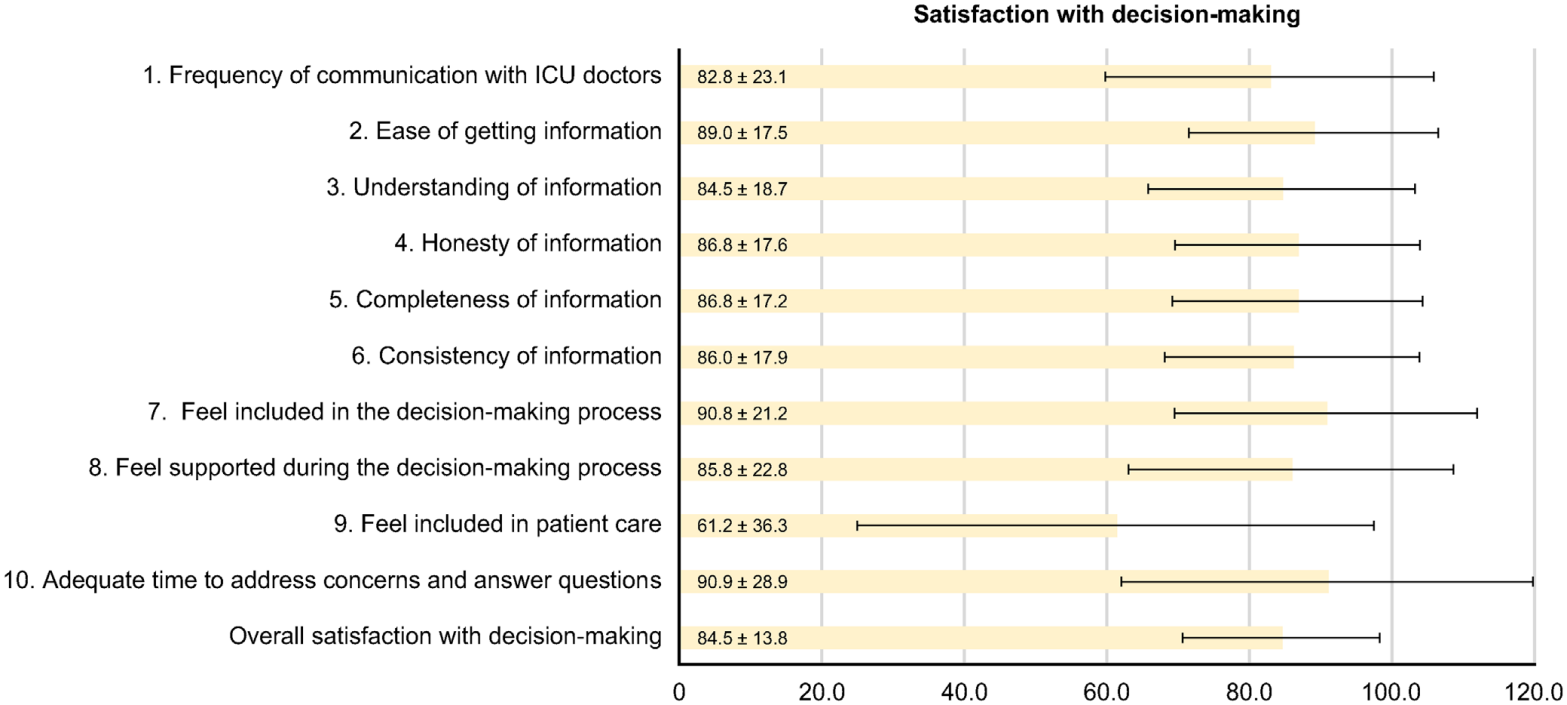
Mean scores and standard deviations for items on the *Family Satisfaction with Decision‐Making for Critically Ill Patients* subscale of the FS‐ICU 24.

The participants' overall score of satisfaction with ICU care (85.7 ± 15.4) was slightly higher than that of satisfaction with the decision‐making process (84.5 ± 13.8). The top five items of participant satisfaction with care were as follows (Figure 1): (1) Concern and caring by ICU staff: The courtesy, respect and compassion your family member (the patient) was given (89.8 ± 14.3); (2) Consideration of your needs: How well the ICU staff showed an interest in your needs (88.5 ± 16.5); (3) Concern and care by the ICU staff: The courtesy, respect and compassion to the family members were given (88.0 ± 16.1); (4) Skills and competence of ICU nurses: How well the nurses cared for the patient (87.0 ± 17.2); and (5) Frequency of communication with ICU nurses: How often nurses communicated about the patient's condition (86.5 ± 18.0). Furthermore, the top five items of Family Satisfaction with Decision‐Making Around Care of Critically Ill Patients were as follows (Figure 2): (1) When making decisions, families have adequate time to address concerns and receive answers to questions (90.9 ± 18.3); (2) Feeling included in the decision‐making process (90.8 ± 21.2); (3) Ease of getting information: willingness of ICU staff to answer family members' questions (89.0 ± 17.5); (4) Completeness of information: regarding the patient's condition and reasons for procedures (86.8 ± 17.6); and (5) Honesty of the information: provided about the patient's condition similar from healthcare team (86.8 ± 17.2).

### 3.4 Factors Associated With Family Satisfaction With the Decision‐Making Process

The total score of satisfaction with care was positively correlated with that of Family Satisfaction with Decision‐Making Around Care of Critically Ill Patients (*r* = 0.808, *p* < 0.001). Table 3 shows that family members with previous ICU experience have higher satisfaction with the decision‐making process (*p* = 0.017) than those without. Regarding demographic factors and correlation with individual decision‐making items, Pearson's correlation analysis revealed that the duration of mechanical ventilation (day) was positively correlated with Item 7 of the Family Satisfaction with Decision‐Making Around Care of Critically Ill Patients (item description: *Feeling of inclusion in the decision‐making process*; *r* = 0.199, *p* = 0.047). Furthermore, the APACHE II score was positively correlated with Item 8 of the same section (item description: *Feel supported during the decision‐making process*; *r* = 0.219, *p* = 0.029).

**TABLE 3:** Differences in the FS‐ICU 24 score between the demographic characteristics of the family members and patients.

Variables	Traditional chinese version of the FS‐ICU 24					
	Overall satisfaction		Satisfaction with care		Satisfaction with decision‐making	
Demographics of families	M (SD)	*p*	M (SD)	*p*	M (SD)	*p*
Sex		0.046*		0.037*		0.091
Female (*n* = 59)	82.8 (13.9)		83.2 (15.4)		82.4 (14.0)	
Male (*n* = 41)	88.1 (12.1)		89.1 (12.2)		87.2 (13.1)	
Previous ICU experience		0.033*		0.092		0.017*
No (*n* = 53)	82.6 (14.5)		83.5 (14.7)		81.7 (15.5)	
Yes (*n* = 45)	88.3 (11.5)		88.5 (14.0)		88.2 (10.5)	
Demographics of patients						
ICU survival		0.019*		0.007*		0.095
Died (*n* = 14)	91.0 (8.9)		93.0 (9.1)		89.1 (10.3)	
Alive (*n* = 86)	84.0 (13.8)		84.4 (14.8)		83.6 (14.2)	

*Note:* FS‐ICU 24, 24‐Item Family Satisfaction in the ICU.

Abbreviations: ICU, intensive care unit; SD, standard deviation.

* *p* < 0.05.

### 3.5 Factors Associated With Overall Family Satisfaction With Care

Regarding the scores of the traditional Chinese version of the FS‐ICU 24, significant differences in demographic factors were observed between family members and patients. The demographic factors influencing the overall FS‐ICU 24 score were the sex of family members, previous ICU experience of family members, and ICU survival of patients. Male family members exhibited higher overall satisfaction (*p* = 0.046) and satisfaction with care (*p* = 0.037) than female family members. Family members with previous ICU experience exhibited higher overall satisfaction (*p* = 0.033) than those without. Furthermore, family members of patients who died in the ICU had higher overall satisfaction (*p* = 0.019) and satisfaction with ICU care (*p* = 0.007) than those of patients who survived. Table 3 presents only statistically significant items. Pearson's correlation analysis was performed to evaluate the factors associated with individual items in the traditional Chinese version of the FS‐ICU 24. No significant correlation was observed between demographic factors and family satisfaction with care items.

## 4 Discussion

This study reported the satisfaction of the family members of ICU patients with care and decision‐making regarding the care for critically ill patients. Furthermore, this study demonstrated the factors associated with family satisfaction. Family satisfaction was investigated using the traditional Chinese version of the FS‐ICU 24. Furthermore, factors associated with family satisfaction with care and decision‐making regarding care for critically ill patients in the ICU were explored. The factors are independent of family members' sociodemographic characteristics at ICU admission, the family–patient relationship, as well as patient characteristics and outcomes [17]. The FS‐ICU is the most widely used, reliable and valid tool for measuring family satisfaction with ICU care [3]. The scores in this study were higher than those in previous studies conducted in Norway [18], South Korea [19] and India [1] but were lower than those in a study conducted in the United States [20]. Thus, despite the quality of care, intensive care in Taiwan should be improved similarly to that in advanced countries.

### 4.1 Factors Associated With Family Satisfaction With Decision‐Making Regarding Care for Critically Ill Patients

The results of this study indicated that previous experience of having family members admitted to the ICU was a factor associated with family satisfaction with the decision‐making regarding care for critically ill patients. This finding supports previous studies demonstrating that previous ICU experience significantly affected overall satisfaction with ICU care [21]. Therefore, family members with previous ICU experience are much more familiar with the routine and regulations compared to those without such experience [22]. Family members without prior ICU experience may experience greater anxiety, as they are unfamiliar with the treatments and equipment. As a result, limited time to understand medical issues could negatively impact their decision‐making satisfaction.

Regarding patient survival during ICU stay, the family members of patients who died in the ICU had a higher satisfaction level than those of patients who survived. These findings are consistent with those of previous research on the overall satisfaction and satisfaction with care for ICU survivors and non‐survivors [21, 23]. Families of ICU patients who died during their stay reported greater satisfaction with physician communication, the consistency and completeness of information, and the support received in decision‐making [21]. Additionally, healthcare teams may place extra emphasis on addressing family wishes as death approaches [24]. Family members who exhibited higher satisfaction with decision‐making regarding care for critically ill patients were more satisfied with care. This finding also indicates that family members require more decision‐making support to achieve high satisfaction with care.

We also found that family members who experienced better support during decision‐making were often caring for patients with longer durations of mechanical ventilation and higher APACHE II scores. This is consistent with previous study findings that greater severity of illness is associated with higher family satisfaction [25]. From some scholars' perspective, families feel sad, helpless and vulnerable when a patient is seriously ill. In such situations, ICU staff play a crucial role in supporting families by spending more time caring for the patient. Therefore, establishing a positive and collaborative relationship between staff and families enhances interaction and involvement, which in turn may increase family satisfaction [26, 27].

### 4.2 Factors of Family Satisfaction With Care

This study demonstrated that sex of the family member, previous ICU experience and patient survival during ICU stay were associated with overall family satisfaction. Although differences between sex and patient survival during ICU stay were observed in satisfaction with care, family members with or without previous ICU experience had different satisfaction levels with decision‐making. In this study, the mean scores for female and male family members were high. Male family members had significantly higher overall satisfaction with care in the ICU. This finding is consistent with previous studies conducted in Saudi Arabia [28, 29]. Gender differences may be influenced by factors, such as communication styles, emotional needs or expectations [24]. In Taiwanese culture, however, most family decision‐makers are male, and they tend to approach information more rationally and have a clearer understanding of the details provided by healthcare teams. A good‐quality communication has been identified as a key factor for enhancing family satisfaction [7]. Future tailor‐made strategies targeting the improvement of family satisfaction should consider sex differences when designing interventions.

### 4.3 Implications and Recommendations for Family Nursing Practice and Research

When patients with critical illness receive treatment in the ICU, their family members may experience significant psychological distress, including anxiety, depression and even acute stress symptoms, which can affect the overall quality of ICU care [4]. During the ICU stay, various factors influence family satisfaction—such as the content of communication, the decision‐making process, nursing care, the ICU environment and the level of spiritual support—with effective communication being the most critical determinant [5, 30]. Enhancing the waiting room environment for family members in the ICU and involving them in patient care are likely to improve family satisfaction with care and decision‐making [22]; thus, healthcare teams may consider planning for the comfort of family members in waiting areas when treating patients with critical illness.

Our study findings support the previous literature demonstrating that the concern and care shown by ICU staff, including courtesy, respect and compassion towards both patients and their families, received the highest satisfaction ratings. This emphasises the central role of family satisfaction in ICU care. To further improve family satisfaction with the decision‐making process, communication should clearly address the etiological diagnosis, disease progression and therapeutic direction for the patient. Furthermore, communication between the healthcare providers and family members should be face‐to‐face using an appropriate tone [4]. The use of structured communication tools, such as patient information leaflets, can provide family members with clearer and more consistent information, thereby facilitating informed decision‐making [31]. For example, various communication skills were adopted in an ICU in Switzerland: After the medical team provided comprehensive communication and explanations to the patient's family, the family's overall satisfaction with the decision‐making process and the quality of patient care in the ICU significantly improved [32]. Favourable communication skills can also reduce the length of ICU stay and medical expenses, which in turn reduce medical resource wastage [33].

In Canada, 17% of families of patients with critical illness required clinical caregivers to visit the patient in person or to follow up on the patients' conditions through telephone interviews after their discharge from the ICU. The main reason was that during their treatment in the ICU, the patients did not have enough time to learn about their conditions, nor did they have opportunities to communicate with the doctors. Furthermore, their family members hoped to express their feelings to the clinical caregivers and seek appropriate consolation after the patients were discharged from the ICU [18]. The implementation of these skills and content in the decision‐making process among family members is likely to increase family satisfaction and enable the family members to feel included in patient care.

In the Taiwanese context, family members' needs and satisfaction can be categorised into five major domains: (1) acquisition of patient‐related information, (2) proximity and companionship with the patient, (3) assurance, (4) environmental amenities and (5) support. Factors influencing family satisfaction include the ICU transfer process, environmental cleanliness, the competence and professional expertise of ICU staff and the nursing staff's attitude towards families [2]. When the attending physician in the ICU responds to family questions during visiting hours, family anxiety is reduced and confidence in the physician's expertise is reinforced, thereby enhancing understanding and satisfaction with ICU care [9, 20]. Moreover, face‐to‐face communication between the attending physician and family members allows the latter to learn about the patient's latest condition and care plans and establish trust in the medical team within a short period. This in turn mitigates the factors contributing to the family's dissatisfaction, such as the nursing staff's unwillingness to explain the patient's condition and the inadequate number and duration of visits [22]. The aforementioned approaches for improving family satisfaction can inform staff training and capacity‐building efforts in Taiwanese ICUs.

This study demonstrates the descriptive results of and factors related to family satisfaction with the decision‐making process and care in the ICU. In addition to providing treatment, the healthcare team must consider the feelings of the family members and achieve the ideal of patient‐ and family‐centred care.

## 5 Limitations

This study has some limitations that should be acknowledged. Although the FS‐ICU was used to evaluate family satisfaction in ICUs, potential selection bias may have been introduced because participants meeting the inclusion criteria were referred by the ICU head nurse. This referral process could have favoured the inclusion of more agreeable or cooperative families, potentially leading to inflated satisfaction scores. Additionally, we excluded participants without a high school education or higher qualification, as our focus was on individuals who required the most support during medical decision‐making. Furthermore, the study was conducted at a single centre. Therefore, a larger and more comprehensive study on family satisfaction in ICUs in Taiwan is warranted to reduce research bias and better reflect the quality of Taiwan's ICU care. Finally, data for this study were collected in 2018. Since then, the ICU environment has undergone a complete transformation because of the COVID‐19 pandemic in 2020; therefore, we recommend that future studies consider the transformation of healthcare following the pandemic, including substantial changes to ICU visitation rules, medical communication methods and the stress levels experienced by family members.

## 6 Recommendations or Implications for Practice and/or Further Research

Family members with previous ICU experience were more satisfied with the decision‐making process. In comparison, ICU clinicians may consider providing clearer and more explicit instructions on healthcare decision‐making to patients and family members receiving ICU care for the first time. Decision aids should be developed for patients to improve healthcare communication, which can help healthcare teams support each other and have the confidence to patiently address their family members' questions for improved care satisfaction and decision‐making.

## 7 Conclusion

Family satisfaction in the ICU is an important indicator of the quality of decision‐making and care. This study assessed the satisfaction of family members of ICU patients to determine whether the quality of ICU care should be improved or whether the treatments or approaches performed in the ICU are worthy of adoption by other medical departments. More decision‐making support should be provided to family members with no previous ICU experience, and the level of satisfaction with care in the ICU should be improved. The results of this study serve as a valuable reference for clinical physicians and nurses regarding their efforts to communicate and share information with patients and their family members, thereby improving the quality of supportive care and family‐centred care in the ICU.

## Funding

The authors have nothing to report.

## Ethics Statement

The study protocol was approved by the Institutional Review Board (IRB) of Taipei Medical University Joint (TMU‐JIRB no.: 201208028, Approval Date: 01/09/2012) and the National Cheng Kung University Hospital (NCKUH‐IRB no.: A‐ER‐106‐291, Approval Date: 01/01/2018).

## Consent

Written informed consent was obtained from the participants after receiving an explanation of the purpose of the study and agreeing to participate.

## Conflicts of Interest

The authors declare no conflicts of interest.

## Data Availability

Because of the sensitive nature of the questions asked in this study, the respondents were assured that the raw data would remain confidential and not be shared.
